# Clinical management of patients with Cushing syndrome treated with mifepristone: consensus recommendations

**DOI:** 10.1186/s40842-020-00105-4

**Published:** 2020-10-29

**Authors:** David R. Brown, Honey E. East, Bradley S. Eilerman, Murray B. Gordon, Elizabeth E. King, Laura A. Knecht, Brandon Salke, Susan L. Samson, Kevin C. J. Yuen, Hanford Yau

**Affiliations:** 1 Private Practice, Endocrinology, Diabetes, and Metabolism, Rockville, MD USA; 2 Metabolic Medicine of Mississippi, Jackson, MS USA; 3 Regional Diabetes Center, St. Elizabeth Physicians, Covington, KY USA; 4 Allegheny Neuroendocrinology Center, Allegheny General Hospital, Pittsburgh, PA USA; 5 Endocrine Associates of Dallas, Dallas, TX USA; 6 Midtown Endocrine Associates, Phoenix, AZ USA; 7 Optime Care, Earth City, MO USA; 8 Pituitary Center, Baylor St. Luke’s Medical Center, Baylor College of Medicine, Houston, TX USA; 9 Barrow Neurological Institute and St. Joseph’s Hospital and Medical Center, University of Arizona College of Medicine and Creighton School of Medicine, Phoenix, AZ USA; 10 Division of Endocrinology, Diabetes, and Metabolism, The University of Central Florida College of Medicine, 13800 Veterans Way, Orlando, FL 32827 USA

**Keywords:** Cushing syndrome, Drug effects, Education

## Abstract

**Background:**

While surgery is the first-line treatment for patients with endogenous hypercortisolism (Cushing syndrome [CS]), mifepristone has been shown to be a beneficial medical treatment option, as demonstrated in the SEISMIC (Study of the Efficacy and Safety of Mifepristone in the Treatment of Endogenous Cushing Syndrome) trial. Mifepristone is a competitive glucocorticoid receptor antagonist and progesterone receptor antagonist that is associated with several treatment effects and adverse events that clinicians need to be aware of when considering its use. The objective of this review was to provide updated clinical management recommendations for patients with CS treated with mifepristone.

**Methods:**

A panel of endocrinologists from the US with extensive experience in treating patients with CS, including with mifepristone, convened as part of a clinical advisory board to develop a consensus on the practical, real-world clinical management of patients on mifepristone.

**Results:**

Comprehensive considerations and recommendations are provided for managing mifepristone-associated effects, including symptoms of cortisol withdrawal, hypokalemia, and change in thyroid function; effects related to its antiprogesterone activity; and rash. Additional management strategies to address concomitant medications and special clinical situations, such as surgery and use in specific populations, are also provided.

**Conclusion:**

Safe and effective use of mifepristone requires clinical judgment and close patient monitoring to ensure optimal clinical outcomes. These consensus recommendations provide useful, practical guidance to clinicians using mifepristone.

## Introduction

Surgical resection of the underlying tumor is first-line therapy for patients with endogenous hypercortisolism caused by Cushing syndrome (CS), regardless of its etiology [1]. Medical treatment options, including pituitary-directed agents, steroid synthesis inhibitors, and glucocorticoid receptor (GR) antagonists, may be used to treat persistent or recurrent disease if surgery fails, or if surgery is not feasible [1, 2]. Medical therapy may also be used as an adjunctive bridge therapy to pituitary radiation while awaiting the effects of radiation [3], and as a preoperative treatment to address the effects of severe hypercortisolism and associated complications [4].

Mifepristone is a competitive GR and progesterone receptor (PR) antagonist approved by the US Food and Drug Administration (FDA) in 2012 to control hyperglycemia secondary to hypercortisolism in patients with endogenous hypercortisolism (Cushing syndrome) who have type 2 diabetes mellitus or glucose intolerance and have failed surgery or are not candidates for surgery. It is not for treatment of type 2 diabetes mellitus unrelated to endogenous CS.

The efficacy and safety of mifepristone in patients with CS was shown in the pivotal 6-month, multicenter, phase 3 SEISMIC (Study of the Efficacy and Safety of Mifepristone in the Treatment of Endogenous Cushing Syndrome) trial (*n* = 50) [5] and in several post-hoc analyses of SEISMIC [6–10]. In SEISMIC, clinically significant improvement was achieved in 87% of patients, as assessed by an independent review board. Because serum cortisol and adrenocorticotropic hormone (ACTH) levels remain unchanged or rise in response to GR antagonism associated with mifepristone, clinicians must monitor the patient’s clinical and metabolic responses to assess efficacy (e.g., body composition, blood pressure, glucose, strength, clinical appearance, and psychiatric and cognitive function) [7]. Clinicians must also use clinical and metabolic responses to assess tolerability and safety. Mifepristone-induced effects include cortisol withdrawal symptoms (fatigue, nausea, vomiting, headache, arthralgia), antiprogesterone effects (endometrial thickening, vaginal bleeding), and changes in thyroid function [5]. Elevated cortisol levels that can occur due to mifepristone’s mechanism of action [8] can saturate the binding capacity of 11β-hydroxysteroid dehydrogenase type 2 (11βHSD2), leading to increased availability of cortisol to stimulate the mineralocorticoid receptor (MR) and resulting in edema, hypertension, and hypokalemia. Clinical improvement following mifepristone therapy may also necessitate adjustment of concomitant medications to prevent potential adverse effects. For example, before starting mifepristone, antidiabetes regimens should be evaluated and modified to reduce the risk of hypoglycemia. Previously published recommendations for the use of mifepristone were based primarily on the clinical experience derived from SEISMIC [1, 11, 12]. Updated clinical guidance is needed on the management of patients treated with mifepristone that reflects the clinical experience gained with this drug since FDA approval in 2012.

In May 2019, a panel of endocrinologists from the US with experience in treating patients with CS, including with mifepristone, convened as part of an advisory board to discuss experiences and reach a consensus on the real-world management of patients on mifepristone. This review summarizes their consensus opinions and is intended to provide clinically relevant suggestions, considerations, and strategies to help practicing clinicians appropriately manage and educate patients on mifepristone therapy, with a focus on safety and tolerability.

## Management of mifepristone’s treatment effects and adverse effects

### Cortisol withdrawal symptoms

**Fig. 1:**
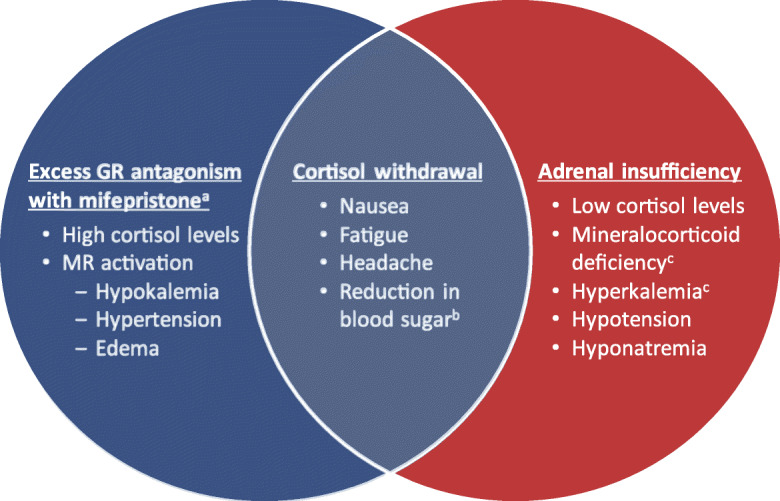
Signs and symptoms associated with excess GR antagonism, cortisol withdrawal, and adrenal insufficiency [8, 15, 16]. ^a^The presence of these signs and symptoms may not always be indicative of excess GR antagonism due to mifepristone and should also be considered in the context of the patient. ^b^Incidence and magnitude may depend on concomitant medications being taken with mifepristone. ^c^Occurs with primary adrenal insufficiency. *Abbreviations*: *GR* glucocorticoid receptor, *MR* mineralocorticoid receptor

Patients with hypercortisolism may experience signs and symptoms of cortisol withdrawal (i.e., nausea, fatigue, headache) during mifepristone treatment. These signs and symptoms, however, and their severity, are wide-ranging and may vary depending on the duration and severity of the hypercortisolism. Of note, some of the signs and symptoms of cortisol withdrawal also resemble those of adrenal insufficiency (AI) [13, 14]. However, several objective signs of severe AI, including hypoglycemia, hyponatremia (primary AI), and hyperkalemia (primary AI) (Fig. 1) [8, 15–17] do not occur during mifepristone treatment [5, 8]. Paradoxically, hypokalemia and/or increases in blood pressure due to MR activation may occur indirectly with mifepristone treatment [5] because of elevated cortisol levels (see also Hypokalemia section, p. 3). These signs and symptoms have been more accurately described as excessive GR antagonism due to overtreatment. (Fig. 1) [8].

Of note, hypokalemia and hypertension can also occur as part of the disease state itself (see the Hypokalemia section below) and may not always indicate excess GR antagonism due to mifepristone. The signs and symptoms should also be considered in the context of the individual patient. In particular, any worsening, persistent, or difficult to treat hypokalemia and/or hypertension may indicate clinically significant excess GR antagonism. If excess GR antagonism is suspected, mifepristone should be temporarily discontinued and rescue therapy with dexamethasone considered [8, 11] at the discretion of the prescribing clinician. Dexamethasone is preferred over other glucocorticoids because of its longer half-life, lack of mineralocorticoid activity, and high affinity for GR [12]. See the Special clinical situations section (p. 9) for dexamethasone dosing recommendations.

Counseling patients on common cortisol withdrawal symptoms before starting mifepristone treatment may help with medication adherence. A brief office or telemedicine follow-up visit within 1–2 weeks may also help reassure patients experiencing cortisol withdrawal that the symptoms are expected, usually transient, and indicative of therapeutic efficacy. See Table 1 for guidance on managing common signs and symptoms of cortisol withdrawal, including headache, arthralgia, fatigue, nausea, and vomiting. Note that treatment with mifepristone can also unmask symptoms of underlying inflammatory and autoimmune disorders such as osteoarthritis, Hashimoto thyroiditis, rheumatoid arthritis, or lupus [18–20].

**Table 1:** Summary of recommendations for the management of some common signs and symptoms of cortisol withdrawal

**Arthralgia**	
Rheumatologic arthralgia	
•May be unmasked during mifepristone treatment	
•Consult with Rheumatology. Consider treatment with nonsteroidal therapies (e.g., biologics/targeted immune modulators)	
Non-rheumatologic arthralgias	
•Recommend treatment with acetaminophen or transdermal preparations (e.g., lidocaine). Avoid systemic/targeted injectable steroids and NSAIDs	
•Nonpharmacological treatments may also be considered, including acupuncture, physical therapy, yoga, weight loss, and TENS	
**Nausea and vomiting**	
Mild nausea and vomiting (i.e., non-emergency setting)	
•5-HT3 receptor antagonists are the first-line treatment. Do not exceed 8 mg of oral ondansetron in order to minimize the potential for drug-drug interactions and side effects (e.g., fatigue)	
•Alternative agents include promethazine or scopolamine patch	
•Consult with pharmacy for potential drug-drug interactions	
Intractable nausea/vomiting	
•If patient is showing signs of volume depletion or is seeking emergency help, temporarily hold mifepristone and give dexamethasone	
**Fatigue**	
Mild fatigue	
•Reassure patients that this is a sign of therapeutic effect and may resolve over time	
Moderate-severe fatigue	
•For fatigue that interferes with the patient’s activities of daily life, recommend additional laboratory assessments, such as thyroid function tests, complete blood count, iron panel, 25-hydroxy vitamin D level, and B12 level	
•Address other comorbidities (e.g., obstructive sleep apnea)	

Note: For intolerable symptoms of cortisol withdrawal, we recommend temporary holding of mifepristone and rechallenge later, restarting at a lower dose

*Abbreviations*: *5-HT3* 5-hyroxytryptamine 3, *NSAIDs* nonsteroidal anti-inflammatory drugs, *TENS* transcutaneous electrical nerve stimulation

Patients, caregivers, and other clinicians should be educated on recognizing the signs and symptoms suggestive of excess GR antagonism, such as hypertension and/or hypokalemia and encouraged to communicate their concerns to the physician managing mifepristone treatment for further assessment. Patients should be instructed to consult with the clinician managing mifepristone when other healthcare providers prescribe new medications, as these medications may interact with mifepristone. Encourage patients to carry a wallet card that contains information on mifepristone, the prescribing physician and pharmacy, the signs and symptoms suggestive of excess GR antagonism (especially hypokalemia), and the general importance of avoiding administration of systemic glucocorticoids for indications other than excess GR antagonism.

### Hypokalemia

**Fig. 2:**
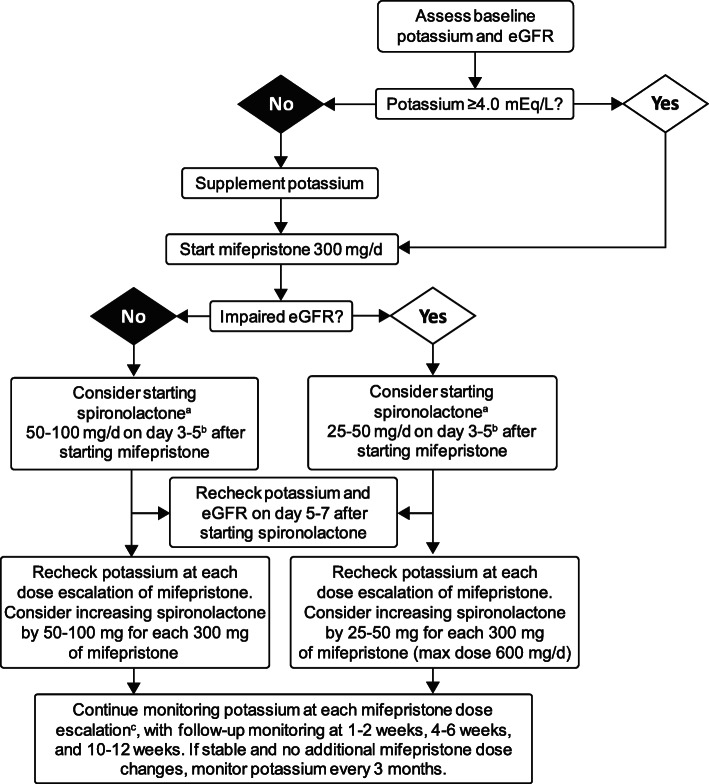
Suggested potassium monitoring and spironolactone dosing algorithm for potassium. ^a^Note that all patients may not need proactive spironolactone. For instance, some patients, particularly those with adrenal disease may not experience substantial increases in cortisol (and associated MR activation) during mifepristone treatment. ^b^MR activation with mifepristone may take several days to occur. Direct patient to call clinician’s office to report when the MR antagonist is started to facilitate appropriate follow-up. If spironolactone is not tolerated, consider an alternative MR antagonist, such as eplerenone. ^c^Check for signs of excess MR activation (e.g., edema, elevated blood pressure). *Abbreviations*: *eGFR* estimated glomerular filtration rate, *MR* mineralocorticoid receptor

Hypokalemia is a frequent manifestation of CS [21, 22] due to excess cortisol saturating the oxidative capacity of 11βHSD2 to inactivate cortisol to cortisone. The excess available cortisol can then act on the MR, leading to MR activation [23]. GR antagonism with mifepristone may lead to further increases in cortisol, particularly in patients with CD, which may potentially exacerbate hypokalemia. In SEISMIC, 44% of patients developed hypokalemia during mifepristone treatment [5]. Hypokalemia should be corrected prior to initiating treatment with mifepristone and potassium levels should be regularly monitored during treatment and dose escalation (Fig. 2) [16].

In addition to checking a baseline potassium level before starting mifepristone, renal function assessment (estimated glomerular filtration rate [eGFR]) and medication review are also advised to identify medications that may alter potassium levels and eGFR (e.g., angiotensin converting enzyme [ACE] inhibitors, angiotensin II receptor blockers [ARBs], loop diuretics, thiazides). For patients on loop or thiazide diuretics, depending on the indication (heart failure or hypertension), the clinician might consider dose adjustment or alternate therapies. Performing a dietary review with the patient and providing recommendations for a low-sodium diet, such as Dietary Approaches to Stop Hypertension (DASH), may also be beneficial [24].

To help further mitigate the risk of developing hypokalemia, a pre-treatment potassium level of at least 4.0 mEq/L is recommended. If pre-mifepristone potassium is < 4.0 mEq/L, we recommend preemptive supplementary potassium prior to initiating mifepristone treatment. Furthermore, we recommend consideration of concomitant treatment with spironolactone. If spironolactone is not tolerated, then the alternative MR antagonist, eplerenone, may be used. See Fig. 2 for suggested spironolactone dosing recommendations. Additional dose modifications may be made based on clinical judgment. In SEISMIC, 28% (14/50) of patients received spironolactone at doses up to 400 mg daily [5].

### Endometrial thickening and vaginal bleeding

Mifepristone is classified as a selective progesterone receptor modulator as well as a competitive GR antagonist. Because of PR modulation, mifepristone can cause endometrial thickening and vaginal bleeding [25, 26]. Among women with available data from SEISMIC, 38% (10/26) experienced endometrial thickening, as detected by transvaginal ultrasound [5, 16]. Although reported primarily among premenopausal women [5], cases of vaginal bleeding with mifepristone have also been reported among postmenopausal women [27]. Therefore, all non-hysterectomized women should be counseled on the risk of potential vaginal bleeding.

Providers should involve gynecology in the management and monitoring of endometrial effects of mifepristone, especially if a transvaginal ultrasound is performed [11]. The endometrial changes associated with PR modulators such as mifepristone are unique and are not indicative of hyperplasia, dysplasia, or malignancy [25, 26, 28]. We recommend that endometrial biopsies be read by pathologists familiar with the endometrial changes associated with PR modulators, to prevent potential misclassification [29, 30]. In the event of bleeding, systemic hormone treatment with progesterone will not be beneficial during mifepristone treatment. For some patients with endometrial thickening, a periodic planned mifepristone holiday, combined with a course of medroxyprogesterone to induce decidual bleeding, may be appropriate [31]. Afterwards, mifepristone may be restarted at the previous maintenance dose. Other options for vaginal bleeding management include dilation and curettage, endometrial ablation, and elective hysterectomy. Implications for future fertility should be discussed with premenopausal patients treated long-term with mifepristone. To our knowledge, there are no available studies on the use of a local intrauterine device to help prevent associated bleeding.

### Thyroid function

**Fig. 3:**
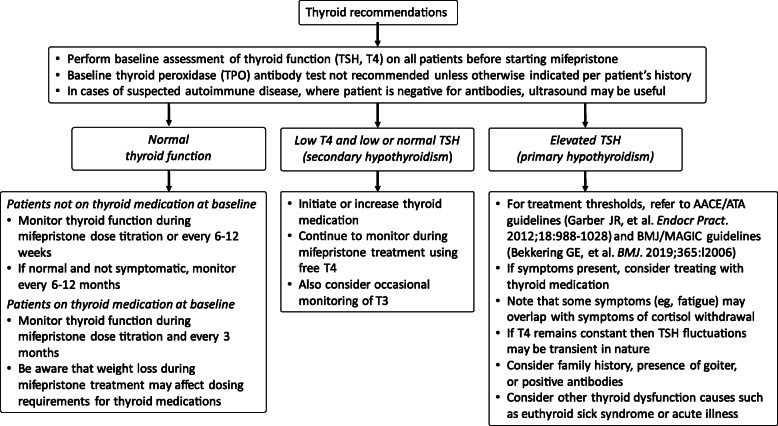
Thyroid assessment recommendations during mifepristone treatment [34, 35]. *Abbreviations*: *AACE* American Association of Clinical Endocrinologists, *ATA* American Thyroid Association, *BMJ* British Medical Journal, *MAGIC* MAGIC (Making Grade the Irresistible Choice) Foundation, *T3* tri-iodothyronine, *T4* thyroxine, *TSH* thyroid-stimulating hormone

Mifepristone use can alter thyroid function [5, 32]. In the SEISMIC trial, 19% (8/42) of patients had reversible increases in thyroid-stimulating hormone (TSH) [5]. TSH increases accompanied by reductions in serum thyroxine (T4) have also been observed within 3 months of long-term mifepristone treatment (up to 40 months) [33]. The mechanisms behind the effects of mifepristone on thyroid function are not yet fully understood and likely differ in patients with primary hypothyroidism versus secondary (central) hypothyroidism. Effects may include increased TSH secretion secondary to hypothalamic and pituitary GR inhibition rather than, or in addition to, reduced thyroid hormone secretion [33]. Pre-existing autoimmune thyroid disease and hypothyroid symptoms may be masked by hypercortisolism [18] and unmasked by mifepristone treatment [18]. If possible, all patients should undergo baseline thyroid function testing before mifepristone is initiated. Regardless of whether a patient is on thyroid medication at baseline, we recommend monitoring TSH and free T4 during mifepristone titration, every 3 months during treatment, and if any signs or symptoms consistent with abnormal thyroid function develop. Monitoring total or free tri-iodothyronine (T3) levels in selected patients may also be of value. Additional monitoring and treatment recommendations are provided in Fig. 3 [34, 35]. Patients with central hypothyroidism may need substantial increases in their thyroid hormone requirement during mifepristone treatment [32]. In a small case series of patients with Cushing disease and central hypothyroidism, patients required a median levothyroxine dose increase 1.83 times the initial dose to achieve normal levels of free T4 [32]. Potential areas for future investigations include further elucidation of the effects of mifepristone on TSH secretion, T4 to T3 conversion, and thyroid-binding globulin levels.

### Rash

There have been reports of rash following mifepristone treatment in patients with hypercortisolism, but published data are limited [16]. Mifepristone-associated rash is characterized as a maculopapular, pruritic rash that typically appears on an extremity and progresses to the trunk. The rash is generally self-limiting and does not reappear upon rechallenge [36].

Should a patient report a rash during treatment, the clinician should assess the patient and rule out anaphylaxis. Other types of rash related to hypercortisolemia, such as cutaneous fungal rash, or autoimmune-related rash (e.g., eczema) resulting from the reduction in cortisol activity should also be considered. Patients should be asked if any new medications have been started that may also be associated with rash. If concomitant treatment with a MR antagonist is planned, starting it 3–5 days after mifepristone is reasonable (see Fig. 2); this may facilitate identification of the causal agent if a rash should develop prior to initiation of the MR antagonist. Have the patient consult with a dermatologist if needed for management of pruritus. Examples of appropriate treatment options include diphenhydramine and hydroxyzine.

## Concomitant medication management

Perform medication reconciliation periodically, especially following visits with outside providers who are not familiar with mifepristone’s mechanism of action, side effects, and drug interactions. Mifepristone prolongs the QT interval in a dose-related manner [16]; therefore, we strongly caution against using mifepristone with other medications that may prolong the QT interval. Advise patients to notify the mifepristone prescriber when any other provider makes a medication change. We also encourage proactive communication with other providers cautioning the use of any medications that affect potassium levels. Specific consensus recommendations to address the management of commonly encountered concomitant medications during mifepristone therapy, including antihypertensives, anticoagulants, and antihyperglycemic agents, are provided in Table 2 [37].

**Table 2:** Suggested recommendations for the management of concomitant antihypertensive, anticoagulant, and antidiabetes medications during mifepristone treatment

**Antihypertensives**	
•If patient is hypertensive at baseline, normalize blood pressure prior to starting mifepristone	
•Recommend MR antagonist (spironolactone) as first-line antihypertensive agent after assessment of renal function (eGFR). Start spironolactone before mifepristone if the patient’s baseline characteristics include hypertension, low or low-normal potassium levels, and/or edema.	
•Use clinical discretion when adjusting other antihypertensive agents, with particular focus on antihypertensive agents (e.g., ACE inhibitors, ARBs, loop diuretics, thiazides) that can affect serum potassium levels and eGFR, particularly when up-titrating spironolactone	
•Be mindful of combination antihypertensive medications. Single agents may be preferable.	
•If the patient is receiving a calcium channel antagonist, note there may be drug-drug interactions between mifepristone and both diltiazem and verapamil. Dihydropyridines are associated with edema, which may be exacerbated by MR activation	
**Anticoagulants**	
Aspirin	
•Low-dose aspirin (81 mg) is acceptable with mifepristone. Do not use NSAIDs due to potential drug-drug interaction.	
Thienopyridines	
•Dose changes not recommended	
Direct oral anticoagulants (DOACS)	
•Among this class, apixaban would be preferred, although there is some potential for drug-drug interaction. Reduce dose per prescribing information [37] and monitor for bleeding events	
•Do not use rivaroxaban due to higher risk of drug-drug interaction	
Warfarin	
•Check international normalized ratio (INR) before starting mifepristone	
•Recommend prophylactically reducing the dose of warfarin by 50% at start of mifepristone	
•Check INR within 48–72 h after starting mifepristone and recheck again after an additional 72 h	
•Check INR following any change in mifepristone dose (within 48–72 h) and recheck again after an additional 72 h	
•After follow-up monitoring and no additional changes to warfarin, have patient continue to monitor INR per anticoagulant clinic guidance	
**Antidiabetes medications**	
Insulin	
•Closely monitor patients for frequent dose adjustments during mifepristone initiation and titration	
•Educate patients to self-monitor glucose levels several times daily, and if possible, arrange for online glucose data sharing	
•Use extreme caution when treating patients with premixed insulins or concentrated insulins (e.g., U-500)	
•Expect a decrease in patient caloric intake with mifepristone (reduction in hyperphagia)	
•Consider proactive adjustment of insulin dose prior to starting mifepristone:	
– If patient is on basal insulin only, consider reducing dose by 10–20%	
– If patient is on basal/bolus insulin, consider reducing bolus insulin by 50% in addition to 10–20% reduction in basal insulin, particularly in patients with severe insulin resistance	
Non-insulin agents	
•Consider stopping sulfonylureas and meglitinides or reducing dose by 50% prior to starting mifepristone because of hypoglycemia risk	
•There is a potential drug-drug interaction between mifepristone and repaglinide	
•Caution against initiating a GLP-1 agonist at same time as mifepristone due to gastrointestinal side effects	
•SGLT-2 inhibitors carry a risk of fungal infection (genital mycotic infection). Monitor patients for volume depletion.	

*Abbreviations*: *ACE* angiotensin-converting-enzyme, *ARB* angiotensin II receptor blocker, *eGFR* estimated glomerular filtration rate, *GLP*-1 glucagon-like peptide-1, *INR* international normalized ratio, *MR* mineralocorticoid receptor, *NSAIDs* nonsteroidal anti-inflammatory drugs, *SGLT-2* sodium-glucose co-transporter-2

### Antihypertensives

Mifepristone treatment may be associated with either a decrease in blood pressure due to inhibition of the effects of hypercortisolism or an increase in blood pressure due to cortisol-mediated MR activation [5]. Therefore, we do not consider it necessary to prophylactically reduce or discontinue antihypertensive medications before starting mifepristone provided patients are closely monitored. In general, when a patient is hypertensive at baseline, their blood pressure control should be optimized prior to starting mifepristone (spironolactone recommended as first-line option). An individualized approach to adjusting other antihypertensives may be needed, with particular attention to antihypertensives that can affect serum potassium levels (ARBs, ACE inhibitors, loop diuretics, thiazides). Monotherapeutic agents are preferred over combination formulations. Other antihypertensives (e.g., beta-blockers, alpha-blockers, calcium channel–blockers) may be indicated based on patient history.

Counsel patients to measure their blood pressure using a home blood pressure monitor during mifepristone treatment and report their results. Patients should be instructed to contact the office in the event of significant blood pressure changes or the development or worsening of peripheral edema. Consider discussing CS medication treatment with co-managing clinicians (e.g., PCP, cardiologist, nephrologist).

### Anticoagulants

Mifepristone increases the concentration of warfarin. Refer to Table 2 for specific recommendations for monitoring and dosing warfarin when using mifepristone. The direct oral anticoagulant (DOAC) apixaban [37] may be used with mifepristone, but caution is required due to the potential for drug-drug interaction. Rivaroxaban [38] would not be a suitable DOAC because it is metabolized primarily by CYP3A (Table 3) [12]. All patients receiving anticoagulants should be counseled on the signs of bleeding and closely monitored.

**Table 3:** Commonly encountered drug-drug interactions with mifepristone

Drug	Interaction^a^	Impact^a^	Recommended approach^a^
**HMG-CoA reductase inhibitors**			
Simvastatin	CYP3A4 metabolism	Increased serum concentration of statins, resulting in increased side effects	**Contraindicated** – switch to rosuvastatin or pravastatin
Lovastatin	CYP3A4 metabolism		**Contraindicated** – switch to rosuvastatin or pravastatin
Atorvastatin	CYP3A4 metabolism (lesser extent)		Switch to rosuvastatin or pravastatin OR lower dose (no more than 20 mg)
Fluvastatin	CYP3A4 metabolism (lesser extent)		Switch to rosuvastatin or pravastatin OR lower dose (no more than 20 mg)
**Opioids**			
Hydrocodone/APAP	CYP3A4 metabolism	Increased serum concentrations of opioids, increasing side effects	Use lowest effective dose and monitor for side effects
Oxycodone			
Tramadol			
Methadone	CYP3A4 metabolism and QT prolongation risk	Increased serum concentrations of methadone, increasing side effects, and risk of life-threatening QT prolongation	Use lowest effective dose and monitor for side effects and EKG changes regularly. Switch to alternative opioid if possible.
Fentanyl	CYP3A4 metabolism	Increased serum concentrations of fentanyl, leading to serious, life-threatening respiratory depression	**Contraindicated** – switch to alternative opioid
**Anticoagulants**			
Warfarin	CYP2C9 metabolism (major) and CYP3A (minor)	Increased risk of bleeding and elevated INR	Increase frequency of INR monitoring. Use lowest effective dose (consider 50% reduction). Monitor after starting mifepristone and with dose titrations.
Rivaroxaban	CYP3A4 metabolism	Increased risk of bleeding	Not recommended
Apixaban	CYP3A4 metabolism (major) and CYP2C9 (minor)	Increased risk of bleeding	Reduce dose of apixaban per prescribing information [37]; monitor for bleeding events
**Antibiotics**			
Azithromycin	QT prolongation^b^	Risk of QT prolongation	Hold mifepristone temporarily while treating infection
Ciprofloxacin			
Levofloxacin			
Moxifloxacin			
Sulfamethoxazole/Trimethoprim			
**Benzodiazepines**			
Alprazolam	CYP3A4 metabolism	Increased serum concentrations of benzodiazepines, increasing side effects	Depending on indication, preferred agents would be lorazepam or oxazepam for anxiety and temazepam for sleep
Clonazepam			
Diazepam			
Triazolam			
Flurazepam			
**Second generation (atypical) antipsychotics**			
Quetiapine	CYP3A4 metabolism (major) and QT prolongation^a^	Increased serum concentrations of antipsychotic, increasing side effects and risk of QT prolongation	Reduce dose and monitor patient and EKG
Risperidone			
Aripiprazole			
Lurasidone	CYP3A4 metabolism (major)	Increased serum concentrations, increasing side effects of lurasidone	Contraindicated with strong CYP3A inhibitors per prescribing information
**Anticonvulsants**			
Phenobarbital	Induce CYP3A4 metabolism (strong)	Decreased serum concentrations of mifepristone	Monitor for clinical endpoints and patient’s improvement. Monitor for changes in seizure activity.
Phenytoin			
Carbamazepine			
Oxcarbazepine	Induces CYP3A4 (weak)		
**Miscellaneous**			
Diltiazem	CYP3A4 metabolism	Increased serum concentrations of mifepristone, diltiazem, and verapamil	Limit doses of both drugs. Start mifepristone at low doses and perform small and careful dose escalations.
Verapamil			
Repaglinide	CYP2C8/2C9	Increased serum concentrations of repaglinide, increasing side effects	Reduce dose or use smallest recommended dose. Monitor blood glucose levels more frequently, especially postprandial levels.
Non-steroidal anti-inflammatory drugs (NSAIDs)	CYP2C8/2C9	Increased serum concentrations of NSAIDs, increasing side effects	Avoid if possible or use smallest recommended dose and monitor for side effects

^a^Applies to all listed drugs in class unless otherwise noted. ^b^For other potential interactions with drugs that prolong the QT interval, check online resources, such as https://crediblemeds.org/index.php/drugsearch

*Abbreviations*: *APAP* acetaminophen, *CYP* cytochrome P450, *EKG* electrocardiogram

### Antidiabetes medications

Mifepristone will increase the risk of hypoglycemia when used in combination with insulins, sulfonylureas, or meglitinides. Patients receiving these antidiabetes medications will need frequent monitoring, and the medications may need to be reduced or discontinued during mifepristone initiation and treatment. Clinicians should expect insulin resistance to improve with mifepristone treatment [5, 9]. Decreased appetite, increased satiety, or nausea during mifepristone treatment may also lower insulin requirements. Extreme caution is necessary when managing patients on premixed insulins or concentrated insulins, such as U-500. See Table 2 for specific proactive insulin dose-adjustment strategies.

When counseling patients, it is essential to emphasize the importance of self-monitoring blood glucose and instruct patients how to down-titrate insulin to avoid hypoglycemia (i.e., reducing or eliminating bolus insulin if limiting caloric intake or skipping meals). The use of continuous blood glucose monitoring devices should also be considered as a method of monitoring glycemic excursions while on mifepristone.

Consider reducing the doses of sulfonylureas and meglitinides by 50% or stopping these medications when mifepristone is started. Other noninsulin antihyperglycemic agents may typically be used with mifepristone with some specific cautions (see Table 2).

### Drug-drug interactions

Mifepristone is metabolized primarily via CYP3A4 and is an inhibitor of CYP3A4 [16, 39, 40]. Mifepristone also inhibits CYP2C8/9 [16]. Therefore, communication with patients and other providers is essential to help prevent potential drug-drug interactions. When using a concomitant strong CYP3A inhibitor (e.g., ketoconazole, itraconazole, ritonavir, clarithromycin), the dose of mifepristone should be limited to 900 mg/day [16]. Additional drug-drug interactions with mifepristone are listed in Table 3. Instruct patients to consult with the clinician and pharmacist managing their mifepristone before starting or stopping any other drug, including over-the-counter drugs and supplements. Clinicians and pharmacists should perform periodic medication reconciliations, particularly following a hospitalization or ER visit.

## Special clinical situations

### Surgery

There is a lack of prospective data to support detailed recommendations for the management of mifepristone in the surgical setting. In general, surgical considerations should include mifepristone’s long half-life (~ 85 h) [16]. Based on the half-life it of mifepristone, it would take approximately 2 weeks to clear from circulation [12]. In the SEISMIC study, serum cortisol levels declined to pre-mifepristone levels at the protocol-specified 6-week post-treatment assessment [5] visit. Additional surgical considerations include the impact of mifepristone on perioperative biochemical measurements, as mifepristone may increase serum cortisol and ACTH levels, and the potential risk of developing symptoms of excess GR antagonism. One must also consider the effects of discontinuing mifepristone and the potential reemerging effects of hypercortisolism on surgical outcomes (worsening glycemic control, hypercoagulability, hemodynamic and electrolyte status and blood pressure changes, potassium level alterations, and impaired postsurgical wound healing). Patients should monitor their blood pressure readings when discontinuing mifepristone.

### Non–hypercortisolemic-related surgery and emergency surgery

If mifepristone is held prior to non**–**hypercortisolemic-related surgery, the MR antagonist may also need to be held. The half-lives of spironolactone and its active metabolites range from 1.4 to 16.5 h [41]. Because of the shorter half-life of spironolactone compared with mifepristone (up to 85 h), spironolactone should be continued until 2–3 days before surgery. If necessary, for patients in an emergency surgical situation, discontinue mifepristone and administer dexamethasone. We recommend a conservative approach to dexamethasone dosing, using 2 mg daily (intravenously or by mouth) for every 300 mg of mifepristone previously administered [11]. Monitor for signs and symptoms of excess GR antagonism (see Fig. 1), as previously discussed. Mifepristone may be restarted 1 week postoperatively, once the patient has normal oral intake, no significant gastrointestinal complaints, and is no longer at risk for postoperative bleeding. Restart mifepristone at the presurgical maintenance dose if tolerated.

### Bridge therapy

Mifepristone has been used as a temporary “bridge” therapy in patients receiving pituitary radiotherapy [42] and as pre-treatment in high-risk patients to reduce perioperative risk and improve postoperative outcomes, such as eliminating or reducing the length of postoperative adrenal insufficiency in some patients [43–47].

Monitoring serum cortisol and ACTH levels over time may be helpful in patients treated with pituitary radiotherapy who are also receiving mifepristone bridge therapy in order to detect the onset of radiotherapy treatment effect, as levels would begin to decrease. Patients must also be monitored for signs and symptoms of AI.

Perioperative management protocols for ACTH-dependent disease vary widely amongst centers and usually include measurements of postoperative ACTH and cortisol levels as indicators of treatment success and risk of relapse. These protocols may need to be modified when managing patients pretreated with mifepristone since mifepristone indirectly increases ACTH secretion from healthy pituitary corticotroph cells. Mifepristone may also be used to control disease following failed surgical procedures while other treatment options are considered.

Patients with unilateral primary adrenal hypercortisolism can also be treated with mifepristone prior to adrenalectomy. Mifepristone affects the hypothalamic-pituitary-adrenal (HPA) axis by antagonizing cortisol activity, which reduces negative feedback at the hypothalamus and corticotrophs, leading to increased ACTH secretion [5, 44, 48]. In patients with unilateral primary adrenal hypercortisolism, the rise in ACTH indicates recovery of previously suppressed corticotrophs [44]. Up to 6 months of pre-treatment with mifepristone may be required to achieve this effect. The increase in ACTH in turn decreases the suppression of cortisol secretion from the contralateral adrenal gland before unilateral adrenalectomy, thereby preventing adrenal atrophy and postoperative AI. This has been shown in a clinical case report [44], where increases in ACTH, accompanied by increases in cortisol and dehydroepiandrosterone sulfate, were monitored as markers of HPA axis reactivation during mifepristone pre-treatment. Additional studies are needed to examine the benefit of GR antagonists as pre-treatment in patients with unilateral adrenal hypercortisolism.

### Infection/sick days

We recommend assessing the patient for signs and symptoms of excess GR antagonism. If no acute signs or symptoms are present, the patient does not likely require supplemental dexamethasone. Clinical judgement is required. Maintain communication with the patient and exercise caution when prescribing antibiotics to prevent any potential drug-drug interactions (see Table 3).

### Exogenous glucocorticoid use

Mifepristone treatment is not appropriate for patients requiring high-dose exogenous glucocorticoid therapies for other underlying conditions. Mifepristone will inhibit the efficacy of these glucocorticoid therapies. Consider switching patients with asthma or chronic obstructive pulmonary disease from glucocorticoid inhalers to long-acting beta-adrenergic or long-acting anticholinergic inhalers. Patients with chronic musculoskeletal pain may experience increased pain when treated with mifepristone due to GR antagonism and may require intensified pain management. If pain medication is needed, check for drug-drug interactions. Consultation with a pain specialist or rheumatologist may be helpful. If a patient on mifepristone subsequently requires exogenous glucocorticoids for comorbidities, for example following a hospital admission, we suggest reevaluating the appropriateness of mifepristone therapy.

### Special populations

In the SEISMIC study, adult patients up to 71 years of age were treated with mifepristone [5]. We recommend the following considerations for the use of mifepristone in older patients (> 65 years):

- Older patients may be prone to develop mild hypovolemia; monitor volume status and GFR, particularly when adding an MR antagonist
- Older patients often receive polypharmacy; monitor for potential drug-drug interactions
- Slower titration may help improve tolerability

Reports of mifepristone use in pediatric patients with CS are limited [31, 49, 50]. In one report, a 16-year-old girl with Cushing disease was treated with mifepristone for 8 years after multiple interventions failed, including transsphenoidal surgery and pituitary radiation [31]. During treatment, she developed endometrial hypertrophy and was given a 2-month mifepristone holiday every 4–6 months, together with a course of medroxyprogesterone to induce decidual bleeding.

Some patients with nonalcoholic fatty liver disease (NAFLD) have shown subtle, chronic dysregulation of the HPA axis that correlates with the severity of liver histopathology, suggesting that hypercortisolism may be implicated in the development of NAFLD [51]. Limited data have shown improvement in liver enzymes in patients with NAFLD treated with mifepristone [52]. Further studies are needed regarding the effectiveness of mifepristone in patients with co-existing fatty liver disease.

### Other clinical considerations

Insulin-like growth factor 1 (IGF-1) levels are elevated in some patients with endogenous hypercortisolism and decrease with GR antagonism [53, 54]. Mifepristone treatment is also associated with reversible decreases in high-density lipoprotein (HDL) cholesterol and HDL particle concentrations along with increases in the efflux capacity of serum HDL (per particle) [5, 55]. The effects of mifepristone treatment on HDL function, thrombo-embolic complications, and cardiovascular outcomes in the setting of CS have not been established [5, 55].

## Conclusion

Mifepristone is a useful medical treatment option for patients with endogenous hypercortisolism, and requires specific clinical considerations and monitoring to ensure optimal patient care and treatment benefit. These consensus recommendations provide practical guidance for the appropriate use of mifepristone and should be used alongside current guidelines for the treatment of endogenous hypercortisolism. Of note, some of the mifepristone clinical management topics discussed do not appear to be dose-dependent, such as those pertaining to thyroid function, endometrial thickening, and vaginal bleeding; therefore, mifepristone dose reductions were not recommended management strategies in those cases.

Close communication with patients during mifepristone treatment is critically important. Evaluation of the efficacy and safety of mifepristone relies heavily on clinical signs and feedback from patients about their symptoms. Therefore, shared decision-making is essential when formulating a mifepristone treatment plan and evaluating its efficacy. Additional assessments may be needed to further differentiate between symptoms of cortisol withdrawal and excess GR antagonism. Communication with other healthcare providers involved in the patient’s care is also critically important, particularly with regards to concomitant medication management.

## Data Availability

Data sharing is not applicable to this article as no datasets were generated or analyzed during the current study.

## Abbreviations

11βHSD2: Hydroxysteroid dehydrogenase type 2
5-HT3: 5-hyroxytryptamine 3
AACE: American Association of Clinical Endocrinologists
ACE: Angiotensin converting enzyme
ACTH: Adrenocorticotropic hormone
AI: Adrenal insufficiency
APAP: Acetaminophen
ARB: Angiotensin II receptor blocker
ATA: American Thyroid Association
BMJ: British Medical Journal
CS: Cushing syndrome
CYP: Cytochrome P450
DASH: Dietary Approaches to Stop Hypertension
eGFR: Estimated glomerular filtration rate
DOAC: Direct oral anticoagulant
EKG: Electrocardiogram
FDA: US Food and Drug Administration
GLP-1: Glucagon-like peptide-1
GR: Glucocorticoid receptor
HDL: High-density lipoprotein
HPA: Hypothalamic-pituitary-adrenal
IGF-1: Insulin-like growth factor 1
INR: International normalized ratio
MAGIC: MAGIC (Making Grade the Irresistible Choice) Foundation
MR: Mineralocorticoid receptor
NAFLD: Nonalcoholic fatty liver disease
NSAIDs: Nonsteroidal anti-inflammatory drugs
PCP: Primary care physician
PR: Progesterone receptor
SEISMIC: Study of the Efficacy and Safety of Mifepristone in the Treatment of Endogenous Cushing Syndrome trial
SGLT-2: Sodium-glucose co-transporter-2
T3: Tri-iodothyronine
T4: Serum thyroxine
TENS: Transcutaneous electrical nerve stimulation
TSH: Thyroid-stimulating hormone
